# The process of contemporary gay identity development in China: The influence of internet use

**DOI:** 10.3389/fpubh.2022.954674

**Published:** 2022-09-06

**Authors:** Aidi Zhang, Nancy R. Reynolds, Chongmei Huang, Simin Tan, Guoli Yang, Jin Yan

**Affiliations:** ^1^Nursing Department, The Third Xiangya Hospital of Central South University, Changsha, China; ^2^School of Nursing, Johns Hopkins University, Baltimore, MD, United States; ^3^Xiang Ya Nursing School, Central South University, Changsha, China; ^4^School of Public Health, Central South University, Changsha, China

**Keywords:** gay identity development, adolescence and emerging adulthood, internet practice, identity confirmation, identity practice, identity coping

## Abstract

**Background:**

Adolescence and emerging adulthood are critical periods for an individual's sexual identity development. The internet has become a primary avenue for gay identity exploration. The purpose of this study was to examine the role of the internet in Chinese young gay male's sexual identity development.

**Methods:**

Qualitative interviews were conducted with 37 gay males aged 16 to 29. Data were analyzed using grounded theory methods.

**Results:**

Three categories of identity development in relation to use of the internet were identified: (1) *Gay identity confirming*, which includes verifying same-sex attraction and connecting the attraction feeling to gay identity through consumption of pornography, Tanbi (boy's love) materials, and searching for information online. (2) *Gay identity practicing*, includes interacting with the gay community to gain understandings of gay subcultures and make close gay friends, engaging in same-sex sexual and romantic relationship development through online group and interpersonal interactions. The internet practice impact on an individual's cognition and behavior by presenting same-sex sexual contact is normal and common, facilitating longing for a romantic relationship, and facilitating relationship development; and (3) *Gay identity coping*, includes increasing self-acceptance of gay identity, gaining a sense of belonging in the gay community, increasing autonomy in sexual and romantic relationship development, and growing consideration of coming out to parents. Young gay males' coping efficacy was gained through the lessons learned from identity practice.

**Conclusion:**

The findings indicate that an individual's internet practice facilitates gay identity confirmation, enriches identity practice, and promotes the transition from self-identification to identity disclosure and integration. Internet practice also threatens to young gay males' sexual risk behavior, emotional hurts from failed romantic relationships. Interventions including sexual identity education and healthy internet use strategies need to be developed tailored to the developmental characteristics.

## Introduction

### Sexual identity and gay identity development

Sexual identity development refers to the process of one's awareness, exploration, interaction, appraisal, and integration of the gender to which one is romantically and/or sexually attracted (1). Adolescence and emerging adulthood (12–29 years) are the critical periods for sexual identity development (2, 3).

Sexual identity is often described as discrete categories including heterosexual, bisexual, lesbian/gay, or as a spectrum from exclusively heterosexual to exclusively homosexual (4). The development of a gay identity is a complex and often difficult process. Linear stage models were originally used to describe the development of a homosexual identity. But with a greater appreciation of the diversity of human sexual experience (5–7), gay identity development has come to be understood as a process with milestones (8, 9). The sequence and timing of each milestone may differ across individuals. (9). Milestones are often used for comparing generational cohort differences, particularly in timing and pacing trends. Studies have found that the younger cohorts report earlier same-sex attraction, self-realization and disclosure than older cohorts, and also report less time between milestones (10, 11).

Young gay males confront challenges during identity development. Meyer's minority stress model posits that distal/external stress (e.g., school/workplace discrimination, stigmatization, harassment, rejection) experienced by sexual minority individuals can generate proximal/internal stress (e.g. internalized homophobia, expectations of rejection, and concealment of sexual orientation) (12). These stressors contribute to lower wellbeing, psychological distress, the risk for depression and suicidal ideation, avoidance of health-seeking behavior, and greater sexual risk behavior related to HIV infection (13–15). The prevalence of young sexual minority depression and suicide is 2.4 to 5 times higher than heterosexual peers and 1.5 to 5 times higher for the prevalence of substance and alcohol use (16, 17). In China, young men who have sex with men account for more than 80% of yearly new HIV infections (18).

### Gay identity development in China

In China, around 30 million males (2.5–5% of the population) self-identify as gay (19). In ancient China, same-sex activity was common among upper-class males. It was regarded as a preference, but not an identity *per se*, as the individuals would participate in a heterosexual marriage and have children. There was also a period of pathologized notion and criminalization of homosexuality, leading to gay group concealment. Li (20) first delineated Chinese homosexual group subculture in the 1980s and 1990s. He described homosexual men as having similar romantic relationship feelings as heterosexuals, but relationships were more unstable and there was a greater engagement in diverse sexual behaviors. Due to a lack of transparency, group members' interactions mostly happened in secret sites like public restrooms, public bathrooms, parks and teahouses. Many members were opposed to heterosexual marriage, but had to engage in marriage to satisfy family and society expectations. During this period, most homosexual members focused primarily on sexual behavior and seldom used the identity word “gay”.

A decade later, Wei (21) found that homosexuality in China went through a transition to become more identity-centric. Although young generations continue to confront difficulties in coming out to family and avoiding heterosexual marriages, younger generations have gained better self-acceptance than the older generations. Young individuals began to use identity-oriented terms such as gay and *Tong Zh*i (“same will,” a contemporary Chinese umbrella term for a non-heterosexual identity. The internet plays a significant role in this change (22).

### Internet practice and gay identity development

There is a saying that the internet has done to Asia's gay and lesbian communities in 5 years of what Stonewall enabled the West to do over 25 years (23). Wei (21) pointed out that young Chinese gay members realize the existence of similar others through the internet. Contrary to the negative stereotypes in the real world, the internet displays normal and positive aspects of the gay community, facilitates community connectedness and self-acceptance (21). The internet is widely used throughout China, with 94.9% of adolescents accessing to it (24). Cyberspace has replaced real space, and become the main field for individual identity exploration, expression, and management. Contrary to hidden gay topics and populations in the real world, the internet offers a way to efficiently gather sexual minority information and community, providing users with controllability and confidentiality (25). Our prior survey showed that three-quarters of Chinese young gay males find their first same-sex romantic or sexual relationship online (26).

Thus, our study aimed at finding out contemporary gay identity development under internet practice. Findings of the study will provide implications of intervention for positive identity, mental and behavioral health among the gay population.

## Methods

Open ended interviews were conducted to gather exploratory data about the influence of the internet on gay identity development in China. Data were collected until information redundancy and data saturation was achieved. Interview data were audiotaped, transcribed verbatim, and systematically analyzed by the investigators using inductive techniques.

### Participants and recruitment

Participants were eligible to participate in this study if they: (1) were between the ages of 16 and 29 years, (2) were born and self-identified as male, self-reported as gay, and (3) were willing to give informed consent. Adolescence and emerging adulthood (10 to late 20s) are the critical periods for one's identity development (27). Besides, based on our prior survey, Chinese young gay males' mean age of identity confirmation is 16.65 ± 3.03 years (26). So, we chose participants aged 16~29 years for our study.

Individuals who expressed willingness to participate in the interviews were screened for eligibility, written or vocal informed consent was obtained from each participant. The recruitment was implemented in Changsha, the capital of Hunan province, located in central south China. Purposive and snowball sampling were employed to recruit participants. For the purposive sampling, the first author acquainted with young gay individuals through a previous qualitative study, invited individuals with diverse ages, cities of birth, and coming-out experiences to participate. Participants were also recruited by posting advertisements in a WeChat group which consisted of over 300 gay members, to recruit individuals willing to attend interviews. For snowball sampling, every participant recruited through purposive sampling was encouraged to introduce the study to his peers who were potential participants. Those who expressed interest in participating were encouraged to contact the researcher.

The recruitment procedure was stopped when information saturation was reached, meaning new data becomes redundant and is not generating new categories. The study was approved by the Institutional Review Board of the Third Xiangya Hospital of Central South University (No. 2016-S180).

### Data collection

The first author conducted all the interviews. An appointment was made with each participant. Aims of the research and participant's rights were explained (confidentiality and rights to withdraw from the study) and informed consent was obtained.

A semi-structured interview outline was used to facilitate the interviews. It included questions about internet practice experience and its influence during each identity milestone according to Savin-William's differential developmental trajectory. The main questions included: When and under what circumstances did you become aware of same-sex attraction/self-identify as gay/develop a first same-sex romantic relationship/ conduct first same-sex sexual contact/first coming out? What were your feelings and coping approaches during the period? How did your internet practice influence this process?

Internet practice in this study refers to finding information, text, video consumption, and interactions with other gay online. Chinese young gay frequently used online tools, including Baidu Tieba, Tencent QQ, WeChat, Weibo, and gay-dating apps. Baidu Tieba is an online community where users may search for a topic of interest forum known as a “bar,” or create one if it does not exist already. Tencent QQ is an instant messaging software service that provides interpersonal and group interactions. A user can search for a group by name and apply to join. Weibo is a Chinese microblogging website, similar to Twitter. WeChat is a Chinese multi-purpose instant messaging app. It provides one-to-one and group text messaging, voice messaging, and video conferencing. A WeChat group consists of at least three members. To join a group, one needs to be invited by a group member. Gay-dating apps, for instance, Blued, is the most frequently used Chinese social networking app for gay men, similar to Grindr, classified as a “location-based real-time” dating application (28).

Thirty-three interviews were performed face-to-face, in the interviewer's office, the participant's home, or in a quiet zone in a park. Four participants were interviewed *via* phone calls since they currently lived outside Changsha city. All the interviews were audiotaped after receiving the participant's consent. After each interview, the participant received an HIV self-testing kit as token compensation for their time. Contact information of the first author and the local CDC was provided in case help was needed if the participant received an HIV-positive testing result.

The median length of the interviews was 68 min, ranging from 42 to 153 min. Participants originated from different parts of China, including nine provinces and one municipality: northern China–Shanxi and Tianjin; eastern China—Shandong; central-southern China–Hunan, Henan, and Guangxi; northwestern China—Gansu and Qinghai; northeastern China—Liaoning and Heilongjiang. The basic characteristics of each participant are displayed in Table 1.

**Table 1:** Characteristics of participants.

**Participants**	**Age**	**Education**	**Urban/Rural birth**	**If the single child of family**	**If coming out to parent/parents**	**If coming out to other people**
Participant 1	20	College student	Rural	Yes	No	No
Participant 2	22	Undergraduate student	Rural	No	No	One friend
Participant 3	24	Bachelor	Urban	Yes	Yes	Classmates, colleagues
Participant 4	19	Undergraduate student	Urban	Yes	Yes	Classmates
Participant 5	23	Undergraduate student	Urban	Yes	Yes	No
Participant 6	23	Undergraduate student	Urban	Yes	No	No
Participant 7	23	Undergraduate student	Rural	No	No	Two friends
Participant 8	24	Undergraduate student	Urban	No	No	No
Participant 9	25	Junior high school	Rural	Yes	Yes	Friends and colleagues
Participant 10	23	College	Rural	Yes	Yes	Friends and colleagues
Participant 11	23	Undergraduate student	Rural	Yes	No	Several friends
Participant 12	29	Bachelor	Urban	Yes	No	Friends and colleagues
Participant 13	20	Undergraduate student	Urban	No	No	One friend
Participant 14	24	Undergraduate student	Urban	Yes	No	Several classmates
Participant 15	23	Undergraduate student	Urban	No	No	No
Participant 16	19	Undergraduate student	Urban	Yes	Yes	Classmates and friends
Participant 17	20	Undergraduate student	Urban	No	No	Older sister, classmates and friends
Participant 18	19	Undergraduate student	Urban	Yes	No	One friend
Participant 19	18	Undergraduate student	Urban	Yes	No	Classmates
Participant 20	25	Senior high school	Rural	Yes	Yes	Relatives and friends
Participant 21	19	Undergraduate student	Urban	Yes	No	Several friends
Participant 22	19	Senior high school	Urban	Yes	Yes	Classmates
Participant 23	28	Bachelor	Urban	Yes	No	Relatives
Participant 24	17	Senior high school student	Rural	Yes	No	No
Participant 25	22	Bachelor	Rural	No	No	Friends
Participant 26	19	College student	Urban	No	No	Classmates and friends
Participant 27	19	Undergraduate student	Urban	Yes	No	Some friends
Participant 28	19	Undergraduate student	Urban	Yes	No	Some friends
Participant 29	19	Undergraduate student	Urban	Yes	No	Some friends
Participant 30	18	College student	Urban	Yes	No	Some friends
Participant 31	20	Senior high school	Urban	Yes	—-	Relatives
Participant 32	18	Senior high school	Urban	Yes	No	Classmates
Participant 33	29	Master	Urban	No	Yes	Classmates and friends
Participant 34	21	Undergraduate student	Urban	Yes	No	Some classmates
Participant 35	25	Bachelor	Urban	Yes	No	Classmates and friends
Participant 36	22	Undergraduate student	Urban	No	No	No
Participant 37	20	Undergraduate student	Urban	Yes	No	Classmates and friends

### Data analysis

The transcribed interviews were coded and thematically analyzed using an inductive technique described by Lofland, Snow and Lofland (29). The first data collected were compared for similarities and differences and coded into categories that reflected participants' experiences. Codes were either: (a) *in vivo* codes, terms used by the participants in the study, or (b) imported codes, codes derived from nursing and/or social science conceptualizations. Initial coding was conducted by breaking each interview material into small sections. Each section was given a brief name that emphasizing the key action or meaning. Focused coding was used to identify the most significant or frequently shown initial codes to reflect most data, elevating initial codes to a higher level. During focused coding, much memo writing was performed to record the researcher's understanding of the data, incidents or actions, to capture the researcher's temporary thoughts on the links and gaps in the codes, and to construct categories by analyzing comparisons, thoughts, and understandings. Each memo was given a specific name. In addition, original quotes were cited in the memo as supports. Constant comparisons were used throughout the whole analysis, between data and codes, data and data, codes and codes, data and memos, to construct focused codes and categories. Similar codes were clustered and given an initial label, thus forming a category. Further analysis produced other categories.

Nvivo 11.0 software was used to assist with the data analyses and administration. Results were displayed with categories, subcategories, and quotes of participants' words as support to illustrate categories. Quality control includes four strategies. First, all the audio-taped interview materials were transcribed verbatim by the first author within 24 h after each interview. To ensure the accuracy of the materials, read through again against the recordings to ensure completeness and accuracy of the transcribed materials. Second, as the first author conducted the coding process, the staged analysis results were supervised by biweekly discussions of the project team. Discussion content includes original interview materials, categories, subcategories, and their supported quotes to ensure the categories' fitness and clarify the next stage of data analysis. Third, after all the material were analyzed and no new category properties were generated, the analyzed results were returned to five participants from the prior interviewees. Their understandings of the categories and subcategories, and if the results illustrate their identity development under internet practice influences were collected to verify results' accuracy and fitness. Fourth, to avoid misunderstanding of original materials, data analysis was conducted in Chinese. The final results were translated into English, including the codes, categories, and supported quotes. The translations were implemented separately by the first and the third author, then form the final version displayed in this manuscript by discussion.

## Results

Three main categories with nine subcategories emerged from the data analysis.

### Gay identity confirming

The internet opens the door for the individual to move from the heterosexual world to the gay world. In the real world, gay topics and populations are hidden, bringing difficulties for the individual to understand his sexual identity and conduct identity exploration. In contrast, there is a wealth of information about sexual identity and gay communities in cyberspace, and it is easy to search.

#### Verifying same-sex attraction

Internet use facilitates one's verification of same-sex attraction. Although a significant proportion of interviewees indicated that they perceived being different from others before internet use, the internet helped them clarify that such differences were related to sexuality.

Some interviewees expressed they felt erotic attractions to men rather than women in pornography materials online. Some interviewees described being attracted to the boy's love stories in Tanbi comics and novels. Tanbi (“dan mei” in Chinese, means the pursuit of beauty). These comics and novels focuse on boy's love (BL), originated from Japan and were brought to China in the 1990's. They were often popular among young women, and many interviewees expressed they knew Tanbi from girl classmates.

*When I was 13 or 14 years old, I came across some porn pop-ups when surfing the Internet. I felt curious and clicked into the windows. Then I realized that I was attracted by the man rather than the woman. After that, I frequently searched and watched homosexual porn videos. (P24, 17 years, high school student)*

*When I was in high school, I knew Tanbi from the girls in my class, I was curious and read some novels from a Tanbi forum in Baidu Tieba. Instead of feeling disgusted about the boy's love like other boys, I yearned to build such a romantic relationship. (P19, 18 years, first year college student)*

#### Connecting same-sex attraction to gay identity

The internet practice promotes one's connecting same-sex attraction to gay identity. P24 joined different online gay groups when he was a teenager, and over time he internalized his group memberships and self-labeled as gay. P19's consumption of Tanbi novels brought emotional resonance that further led to identity resonance.

*I found a gay bar on Baidu Tieba. It provided the contact information of QQ gay groups. When I joined the groups, I found more information and joined more groups. The groups opened another world for me. I observed their chatting and gradually interacted with them. I was young, my relationship network was small, and gay groups occupied a large part of the network. I naturally think I'm a group member and I'm gay. (P24, 17 years, high school student)*

*After the college entrance examination, I got more free time. I read more Tanbi novels and joined a Tanbi bar on Baidu Tieba. The boy's love stories made me laugh and cry with the storylines up and down. I resonated with their story and thought I might be gay. (P19, 18 years, first year university student)*

Some participants expressed that although they had realized same-sex attraction since early childhood, it was not until they got access to the internet that they searched for the answer to their attraction and found gay identity as the answer.

*When I was 15 years old, I got access to computers and learnt how to surf the internet…I knew I was attracted to boys rather than girls since I was very young. One day, I was so curious that I searched “why I have feelings for boys.” The word “gay” came out, along with some gay groups' contact information. I got the answer at that moment. (P9, 25 years, office worker)*

### Gay identity practicing

Internet practice deepens one's involvement in the gay community. Individuals find information about gay groups and get access to gay community easily through the internet. The interviewees expressed that with their interactions in the groups, they learned about the gay community subculture, and built their relationship network. With the help of online group resources and gay-dating apps, the interviewees explored sexual and romantic relationship development.

#### Interacting with gay community

First is one's gaining knowledge of gay community. Most interviewees expressed they initially learned about the existence of gay people from Baidu Tieba, then got information about online gay groups (from Tencent QQ group) and became a group member. Each group contains hundreds to thousands of gay members. Group members hardly know each other offline, and their online group interactions vary from silent observers to active chatters. Individuals gained understandings of gay subculture from the group conversations and information sharing, including gay specialized words, sexual roles, sexual values, sexual dating behaviors, shared interests, etc.

*It was from the gay QQ groups, I learnt the meaning of 1 (dominant), 0 (submissive) and 0.5 (versatile); gong (*攻 *, dominant) and shou (*受 *, submissive); chu gui (*出柜 *, out of the closet, means coming out), etc*. *In most of the groups, members' conversations are about sexually explicit materials sharing, sexual dating. But I also joined some groups where people reject sexual dating, they talk about career plans and long-term relationships. I joined in different groups and learnt different values. (P32, 18 years, high school graduate)*

Second is one's building of one's own relationship network in the gay community. As individuals spend more time interacting with online gay groups, they make friends and form relationship networks, which they call small groups. The small groups, often in the form of WeChat groups, facilitate members' active online and offline interactions. Small group interactions deepen their friendship, integrate into daily lives, and become an alternative to the prior QQ group (big group) interactions.

*I got involved with a gay WeChat group of 20 members, introduced by a friend. The members are all excellent. I became close friends with three of them, a lawyer, a college teacher, and a fashion lover. We had lots of fun together. (P11, 22 years, fourth year university student)*

*I met three friends at an offline party organized by the administrator of a gay QQ group. We are similar in age, and our universities are close to each other. In the past 2 years, the four of us often hung out together on weekends. We became very good friends. I rarely hang out with anyone other than the three of them. (P21, 20 years, second year university student)*

#### Engaging in same-sex sexual practice

Initially, forming the value that same-sex sexual behavior is normal and common. Individuals frequently encounter sexual dating conversations and sexually explicit materials in online gay communities. They gradually became desensitized to same-sex sexual behavior, and perceived it as normal rather than weird, common rather than rarely seen.

*I was 14 years when I first entered online gay groups… I watched the group members flirting, sharing porn images and videos. They are always sending sex dating messages in the groups.… I was so young and watched them chatting in groups everyday. I naturally think gay sex is relatively normal. (P24, 17 years, high school student)*

*Even the one who is excellent in careers and behaves decently, who I used to regard as a role model, 1 day, I noticed he had lots of sexual material on his computer, and he also engaged in sexual dating behavior. So, sex is common among gays. (P32, 18 years, high school graduate)*

Next is promoting the onset and practice of sexual contact. The sexual dating conversations and sexual materials in online gay communities encourage young gay men's curiosity about sex. In online groups, it is popular for two gays to go from online chatting to offline meeting, called *mian ji* (面基, means offline dating with a gay). This can significantly increase the probability of sexual contact. Some interviewees may actively seek sexual partners on the internet. And for interviewees who do not plan to have sex when initiating online interactions, they rarely refused the other's sexual demands in offline dating.

*I entered the online gay community very early. Many members sent sex dating messages in the groups, like “I'm a 1, 180 cm, 75 kg, located (the place name), and want to find a 0.” … I was curious about sex after seeing so many things in the group. Then I arranged a date, and my first time (sexual behavior) happened when I was 14 years old. (P24, 17 years, third year high school student)*

*I had two offline dating experiences..*. We chatted online for a while in the second dating, and met offline *He took me to his home, we had sex. Although I didn't want to have sex and expressed refusal at first, I didn't strongly refuse. I felt regret. (P21, 19 years, second year university student)*

#### Engaging same-sex romantic relationship

Participants described the desire for a romantic relationship. With the consumption of Tanbi novels, films and TV series, and other gay members' sharing of their romantic stories, participants expressed the desire for same-sex romantic relationships.

*I watched boy's love stories on Baidu Tieba during high school. I brought myself into the stories' context, became happy when the story was sweet and cried when their love was confronted with challenges. I want to have a romantic love of my own. (P2, 22 years, fourth year university student)*

*I followed a gay couple's Weibo, they adopted a son. The boy is now in his teenagers. They share their daily life and stories. I envy them and long to find a same-sex partner, develop a family like them, and spend the rest of our lives together. (P16, 19 years, second year university student)*

Second is initiating relationship development. Online gay groups and gay dating apps were the main tools for the interviewees to find romantic partners. Some interviewees played as observers in the online groups to find members who wanted romantic relationships rather than purely sexual relationships, and initiated interactions with the potential partners. Others expressed their searching for potential romantic partners through gay dating apps, based on the geographic location function of the apps to find people nearby to chat.

*After the college entrance examination, I thought I was old enough and had strong feelings about falling in love. I joined several online gay groups and met one person in a group who expressed his desire to find a boyfriend. I responded to him, and built my first romantic relationship. (P2, 22 years, fourth year university student)*

*I really want to find a boyfriend. I'm already in college, many of my heterosexual classmates have a boyfriend or a girlfriend. But I don't want to join in online gay groups since they are mainly looking for sex. So, I use the app Blued to find people from nearby universities to chat. I want to try my luck and see if I can find one. (P27, 19 years, second year university student)*

### Gay identity coping

With increased knowledge of sexual identity, interactions with the gay community, and experiences of sexual and romantic relationships, the interviewees adapted to their gay identity, becoming more confident in identity coping in different contexts.

#### Increasing acceptance of gay identity

Individuals continuously internalize a gay identity during the process of identity practice, which includes community interactions, sexual and romantic relationship practice. They become adapted to gay identity over time.

*This is my fourth year since I joined the gay community. I have gone from learning what's gay in Baidu Tieba to chat with other gays in various QQ and WeChat groups, and I frequently use gay-dating apps, etc. I got to know the group well, I had sex and romantic relationships. Now, I'm very used to my gay identity, and familiar with behaving as a gay in cyberspace. (P21, 19 years, second year university student)*

Interviewees mentioned that they perceived most members of the gay community are accepting of their gay identity. The members have good careers and normal lives. The perceptions relieved interviewees' identity concerns and improved self-acceptance.

*In the gay QQ groups I joined, I saw many members sharing their lives and work. It seems that they have as promising careers and lives as heterosexuals, the gay identity didn't cause them much loss. So, being gay is not a big deal. (P24, 17 years, third year high school student)*

#### Gaining sense of belongingness in the gay community

The sense of belonging comes from the existence of the gay community with a large number of members. Interviewees expressed the trajectory from the initial perception of being different, awareness of same-sex attraction, to participation in online gay groups. One's feelings during the trajectory went from being alone and weird to belonging to a community with similar others.

*I used to see myself as a weirdo since no one around is like you. But after interacting with other gays, I realized there are so many people similar to me, my problems are similar to theirs. If we all face the problems, life is not as hard as I thought. (P32, 18 years, high school graduate)*

However, as most members in the online groups are unknown to each other, young members may experience sexual, emotional deception, or financial fraud, which can damage their sense of belonging.

*I met one person on the gay-dating app, he made a good impression on me. But it ended that I was frauded and lost 1,800 Chinese yuan (about 250 dollars)... I realized the community was not as good as I thought. I don't use gay apps as much as before. (P37, 19 years, second year university student)*

In contrast, interviewees expressed that after building their own relationship network with close gay friends, they perceived friendship and a deeper sense of belonging. Most of the interviewees valued the development of the “small groups.” One interviewee expressed that under the conservative social context, it is difficult to gain acceptance by the society and family toward his gay identity, but members in the small group would support each other. The group plays a family role.

*I got an operation last summer and was hospitalized. My straight friends just came to see me and left for their internships. But my three gay friends came to take care of me in turn for about 10 days. They are my real friends rather than my straight friends. (P11, 23 years, fourth year college student)*

*I value that a gay person must have a small group with friends who can care for each other, just like a family. The friends may give you more support than your real family. Without such a group, I would probably end up alone. (P6, 23 years, fourth year college student)*

#### Increasing autonomy in relationship development

Increasing autonomy is first formed through one's relationship development beliefs based on sexual and romantic relationship experiences. Some interviewees believe that although internet is the primary way for interpersonal relationship development, this approach is unreliable in many cases. Some interviewees felt disappointed when reaching out to offline dating, and the online to offline meetings which turned into non-consensual sex also made them feel regret.

*I've had two offline dating experiences through Blued app, but both times I felt disappointed. We had good conversations on the app, but I felt uncomfortable when we actually met in person. The person was not as good as his introduction in online personal files. Finding a boyfriend through the gay-dating app is unreliable. (P16, 19 years, second year university student)*

*I had two offline dating experiences. I hoped to develop a romantic relationship but ended with unwanted sexual contact. The failed experiences taught me that it's hard to succeed using gay dating apps to develop romantic relationships. (P21, 19 years, second year university student)*

Some interviewees believe that one should pursue the chance for a romantic relationship in time, and meanwhile, be ready for a relationship that may not last.

*I had three romantic relationships, two started on Weibo and one on a dating app. The three experiences taught me that if you have feelings for someone, don't waste your time, just pursue it. And if your relationship does not last long, don't be so upset. (P34, 21 years, third year university student)*

Second, involves increasing one's controllability of online interpersonal interactions. The interviewees described becoming more confident in selecting whom to interact with online. College students preferred to find people from nearby universities, believing that college students are more focused on romantic relationships rather than casual sex, and pay attention to safety and HIV prevention, leading to a higher possibility of a successful relationship. Moreover, interviewees expressed they gained higher self-efficacy in distinguishing whether the other person chatting online was aimed at a sexual or romantic relationship. And they would stop the interaction once evaluating that the online conversations were not in congruence with his relationship development preferences, avoiding further loss.

*I always find people from nearby universities to chat with. They have received a good education, and I'm familiar with the environment, which is safe for me. I also wrote in Blued's personal file that “I don't accept casual sex,” to keep me away from those who just want sex. (P32, 18 years, high school graduate)*

*If someone suddenly talks to me, and it is all about offline dating or flirting, I can tell he is not the one I want to find, he just wants sex. (P21, 19 years, second year university student)*

#### Increasing considerations of coming out decision and strategy

Eleven interviewees joined the PFLAG online group in their city. PFLAG means Parents, Family and Friends of Lesbians and Gays, with chapters in different cities. The chapters often use WeChat groups for membership joining and interaction. Coming out to parents is one of the main topics of conversation in the group. Some members ask for advice or put forward concerns about coming out, and others would share their own coming out stories and provide advice. The PFLAG group also organizes offline activities, sometimes live-streaming their activities through dating apps.

*In PFLAG WeChat group, members often seek help about their problems when coming out to their parents. Other members who have gone through coming out process would share their experiences and offer advice. The conversations encouraged me to think about the coming out issues I may encounter in the near future. (P30, 18 years, first year college student)*

In the PFLAG group, the parents of gays and lesbians shared their psychological journey of accepting their children. Interviewees expressed that from the experience of other parents in PFLAG, they understood parents would also go through a difficult journey, and they need to prepare for their parents' possible reactions after their coming out.

*I watched a mom from PFLAG share her story with her gay son on Blued live-streaming. She took 10 years from nearly breakdown to full acceptance of her son's gay identity. It was a long and complex journey. I realized that my parents may also need a long process to accept me if I come out, and I need to be prepared. (P16, 19 years, second year university student)*

Interviewees expressed that the PFLAG group is an important helping resource. They regarded the parents with gay children in PFLAG group as resources helping them to persuade their parents when coming out. Interviewees also expressed their desire to bring parents to the PFLAG group's activities and improve their parents' understanding of the gay community and gay identity.

*I really hope that 1 day I can bring my mom to the PFLAG's activities. I believe if she knows other parents' stories, she will be more understanding and accept me. I also told a mom in the PFLAG that I hope she can help me communicate with my parents when I come out in the future. (P29, 19 years, second year university student)*

## Discussion

Contemporary Chinese gay identity development is a process that is facilitated by Internet use. This study used qualitative methods to explore the developmental process. Three categories were identified, gay identity confirming, gay identity practicing, and gay identity coping. These three categories describe a process from preliminary exploration to a deeply evolved gay identity.

The process covered Savin-Williams' (9) milestones for gay identity. This indicates that the nature and content of gay identity process have not significantly changed with online practices. Overall, the internet practice facilitates individual's sexual identity development. In the direct pathway, the internet provides young gay males with an extensive resource pool. Participants get information, learn gay subculture, observe and imitate other's thinking and behaving. In the socially mediated pathway, individuals join online gay community, interact with other members, get their views, and facilitate identity practice and coping efficacy.

The internet practice facilitates individual's sexual attraction awareness and identity confirmation. Unlike the phenomena described by Li and Wei (20, 21), where individuals who had same-sex sexual behavior but were not aware of or identified as gay identity, the online information directly presents the interconnectedness of the two elements, leading to one's self-label as gay. Our research found that Tanbi product consumption online facilitates adolescents' early understanding and awareness of same-sex attraction. Unlike hidden gay topics in mainstream mass media, the flourishing of Internet Tanbi drama, which talks about the culture of Boy's Love (BL) and popular among sexual minority as well as heterosexual adolescents creates a chance for sexual minority culture to officially go into the public view (30). Pan's research pointed out that Tanbi products potentially impacted on Chinese adolescents' sexual orientation. Our previous survey also showed adolescents who have more consumption of Tanbi drama and novels are more likely to self-identify as gay/lesbian. Although young males who perceive same-sex attraction tend to seek Tanbi content more than others, Tanbi drama delineates beautiful boy's love and may guide inexperienced adolescents longing for a same-sex romantic relationship.

Cyberspace provides individuals with a place for identity practice and enriches relationship experiences. First, individuals gradually integrate gay identity into their lives. Individuals are able to interact with groups online freely, enrich sexual and romantic relationship experiences, which supports their getting used to thinking and behaving as a gay in cyberspace. As the interviewees said, “The gay community is as deep as an ocean. Once you enter the ocean, you gradually become a part of it and it is hard to get out.”

Second, identity practice is offered through community and interpersonal interactions online. Two levels of community interaction were found, one's interactions in the online group with a large number of members, which we named “big group” interactions; and one's relationship network with close gay friends, which we named “small group” interactions. The prior one allows individuals to quickly learn about gay subcultures, including gay-specific used words, values, behavioral patterns, etc., and keep noticing what's happening in the community. Other scholars' who explored gay individuals' use of Facebook and Grindr showed similar findings (31–33). Whereas, the ubiquitous sexual dating atmosphere in online gay community leads to individual's disappointment toward the group. The small group is a kind of refined network of gay acquaintances, members in the group hold similar values. The similarity, consistency, and closeness contribute to social interactions (34), effectively reduce the disappointment dilemma in the big group. Therefore, during the later phase of one's gay identity development, small group interactions may play a more critical role. Chinese people focus on social relationship, especially the acquaintance network. Participants expressed the small group some kind of a substitute home. Compared with their original home and friend circles consist of heterosexual members, they gain more belongingness and emotional support from close gay friends.

The internet practice makes cognitive and behavioral influences on young gay male's sexual and romantic relationships. The influence on sexual cognition manifests in perceptions and attitudes toward sexual behavior, same-sex sexual contact, and sexual dating (35). Individuals are consistently exposed to sexual topics and sharing of sexually explicit materials, forming the views that sex is convenient, same-sex sex is normal and prevalent, sexual dating is a popular behavior among gay community. The cognition leads to gay people's curiosity and pursuit of satisfying sexual needs. The influence on romantic relationship cognition is mainly presented as overly perfect expectation of relationship from delineation of Tanbi drama. This may result in two problems, one is the high relationship expectation may lead to easily break up when a relationship not satisfy his imagines. Another problem is that adolescents may lack of ability to distinguish sexual and romantic relationship clearly, they might mistake sexual dating as the start of a romantic relationship, which brings potential sexual risks.

Internet practices enhance young gay males' identity coping efficacy. Our study showed that identity coping was reflected in four aspects: self-acceptance, sexual and romantic relationships, gay community involvement, and identity disclosure. They reflect the significant social relationships for gay identity development. Young gay male's efficacy was enhanced by long-term exposure to community information and identity practice, promoting the identity deepen process. Meanwhile, there is also a problem: since most young gays' identity exploration by personal experience, their efficacy often improved after negative experience, emotional hurt, and sexual risk behavior. Thus the early period of identity development is also the period of high-risk psychosocial and behavioral health problems. Early education and support are essential. In addition, young gay males' internet practice contributes to the consideration of coming out. Previous studies by Chinese scholars have shown that cyberspace provides space for gay identity expression and group consciousness. Still, at the same time, gay groups' interactions tend to happen among the inner gay community and avoid interactions with the heterosexual mainstream (36–39). Chinese gay identity development during the past 30 years changed from a lack of identity awareness to identity-centric, and to good self-identification but concealment from others (40). Our study further found that contemporary young gay males accumulate strategies for coming out to parents during internet practice, they become more emphasis on social identity such as family acceptance. The internet provides them with a field gaining resources and testing water.

### Implications for intervention

The influence of internet runs through the whole process of gay identity development. For identity confirmation, scientific knowledge of sexual identity should be provided, including what sexual identity is; the relationship between sexual attraction, sexual orientation, and sexual behavior, and recognition of the influence of online pornographic and Tanbi materials on individual sexual cognition (41). Besides, homosexuality is a normal phenomenon, but premature and risky sexual behavior is not encouraged. The relationship between high-risk same-sex sexual behavior and HIV should be given.

For identity practice, intervention needs to promote healthy interpersonal and group interactions. First is to improve young gay males' recognization of the internet practice impact pathways (i.e., how individual's online interactions affect his understandings of gay community, sexual contact and romantic relationship, and further affect his mental and behavioral health). Second is to clarify that although there are high incidences of casual sex and substance use in gay groups, the behaviors are unhealthy and dis-encouraged. Third is to promote young gay males' establishment of healthy relationship development values. A healthy relationship is the integration of intimacy, sex, and commitment. A relationship that lacks commitment, or separate affection from sex, will lead to unsustainable relationships.

For identity coping efficacy, the efficacy is often gained after the individual has already experienced mental and behavioral health threats. Therefore, interventions should target the possible negative experiences (e.g., internalized homophobia, sexual health threats, emotional harms, unhealthy group interactions, etc.) and be implemented early, especially during adolescence. Moreover, family coming out has become a significant conflict for contemporary young gay. Therefore, interventions should also provide practical gay child-parent communication skills.

Finally, interventions should include healthy internet use strategies, and use the internet as a venue for intervention implementation.

### Limitations

First, most of the participants in this study were current college students. The high school period is an essential stage for gay youths transferring from identity confirmation to sexual and romantic relationship practice. Although we asked all participants to recall experiences during high school, here may still be missing information. Second, only young gay males were involved in this study, future studies may extend to lesbians, transgender individuals, bisexuals and other sexual minorities to provide more evidence on the internet's influence on sexual identity development, and to explore differences between different sexual minorities. Third, in this study, about 75% of the interviewees came from urban. Although the young generation has relatively high internet access, and we used information saturation as the principle for data collection, considering young gay males from urban and rural areas might have different growth environments, holding different values, which may still lead to information bias. Fourth, this study illustrated qualitative findings of the gay identity development under the influence of internet practice. A quantitative approach is necessary to describe internet use and its correlations with identity status. We also conducted a quantitative survey, findings will be presented in another manuscript.

## Conclusion

This study explored contemporary Chinese gay identity development under the influences of internet practice. The process is manifested by a shift in venue from real space to cyberspace, the internet provides individuals with an extensive resource pool. In this field, individuals can quickly connect with gay identity and gay community in a controllable manner (without personal information exposure); enrich their identity practice experiences, especially community interactions, sexual and romantic relationship development; and thus enhance their identity coping efficacy. However, we also noticed the potential threats to sexual and mental health through the internet's impacts on individuals' sex cognition and behavior. It is necessary to provide interventions that are consistent with these developmental characteristics. In addition, contemporary gay groups are shifting from self-identification and in-group interactions to identity disclosure and integration, presented as intergenerational evolution of identity development.

## Data availability statement

The original contributions presented in the study are included in the article/supplementary material, further inquiries can be directed to the corresponding author/s.

## Ethics statement

The study was approved by the Institutional Review Board of the Third Xiangya Hospital of Central South University (No. 2016-S180). The patients/participants provided their written informed consent to participate in this study.

## Author contributions

AZ contributed interviews conducting, data analyzing, and manuscript draft writing. JY contributed design of the study and the principle investigator of the project. NR, CH, ST, and GY contributed data analysis, translation, and manuscript drafting. All authors contributed to the article and approved the submitted version.

## Funding

This study was supported by the National Social Science Fund of China (Grant No. 18BSH033), and Hunan Natural Science Foundation (Grant No. 2022JJ40737).

## Conflict of interest

The authors declare that the research was conducted in the absence of any commercial or financial relationships that could be construed as a potential conflict of interest.

## Publisher's note

All claims expressed in this article are solely those of the authors and do not necessarily represent those of their affiliated organizations, or those of the publisher, the editors and the reviewers. Any product that may be evaluated in this article, or claim that may be made by its manufacturer, is not guaranteed or endorsed by the publisher.
